# Complications and outcomes of tubeless versus nephrostomy tube in percutaneous nephrolithotomy: a systematic review and meta-analysis of randomized clinical trials

**DOI:** 10.1007/s00240-022-01337-y

**Published:** 2022-06-08

**Authors:** Vineet Gauhar, Olivier Traxer, Esther García Rojo, Simone Scarcella, Maria Pia Pavia, Vinson Wai-Shun Chan, Eugenio Pretore, Marcelo Langer Wroclawski, Mariela Corrales, Ho Yee Tiong, Ee Jean Lim, Jeremy Yuen-Chun Teoh, Chin-Tiong Heng, Jean de la Rosette, Bhaskar Kuman Somani, Daniele Castellani

**Affiliations:** 1 Department of Urology, Ng Teng Fong General Hospital, Singapore, Singapore; 2 Department of Urology Hôpital Tenon, Sorbonne University, Paris, France; 3 Department of Urology, Hospital Universitario HM Sanchinarro, HM Hospitales and ROC Clinic, Madrid, Spain; 4 Faculty of Medicine, School of Urology, Università Politecnica delle Marche, Ancona, Italy; 5 School of Medicine, Faculty of Medicine and Health, University of Leeds, Leeds, UK; 6 Hospital Israelita Albert Einstein, BP-a Beneficência Portuguesa de São Paulo, Sao Paulo, SP Brazil; 7 Faculdade de Medicina do ABC, Santo André, SP Brazil; 8 Department of Urology, University Surgical Cluster, National University Hospital, Singapore, Singapore; 9 Department of Urology, Singapore General Hospital, Singapore, Singapore; 10 Department of Surgery, Faculty of Medicine, The Chinese University of Hong Kong, S.H. Ho Urology Centre, Hong Kong, China; 11 Department of Urology, Medipol Mega University Hospital, Istanbul Medipol University, Istanbul, Turkey; 12 Urology, University Hospitals Southampton NHS Trust, Southampton, UK; 13 Urology Unit, Azienda Ospedaliero-Universitaria Ospedali Riuniti di Ancona, Faculty of Medicine, School of Urology, Università Politecnica delle Marche, Via Conca 71, 60126 Ancona, Italy

**Keywords:** Percutaneous nephrolithotomy, Kidney stone, Percutaneous nephrostomy, Ambulatory surgical procedures

## Abstract

We aimed to perform a systematic review of randomized trials to summarize the evidence on the safety and stone-free rate after Tubeless percutaneous nephrolithotomy (PCNL) (ureteral stent/catheter, no nephrostomy) compared to Standard PCNL (nephrostomy, with/without ureteral stent/catheter) to evaluate if the tubeless approach is better. The inverse variance of the mean difference with a random effect, 95% Confidence Interval (CI), and *p* values was used for continuous variables. Categorical variables were assessed using Cochran–Mantel–Haenszel method with the random effect model, and reported as Risk Ratio (RR), 95% CI, and *p* values. Statistical significance was set at *p* < 0.05 and a 95% CI. 26 studies were included. Mean operative time was significantly shorter in the Tubeless group (MD—5.18 min, 95% CI − 6.56, − 3.80, *p* < 0.00001). Mean postoperative length of stay was also significantly shorter in the Tubeless group (MD—1.10 day, 95% CI − 1.48, − 0.71, *p* < 0.00001). Incidence of blood transfusion, angioembolization for bleeding control, pain score at the first postoperative day, the number of patients requiring postoperative pain medication, fever, urinary infections, sepsis, perirenal fluid collection, pleural breach, hospital readmission, and SFR did not differ between the two groups. Incidence of postoperative urinary fistula was significantly lower in the Tubeless group (RR 0.18, 95% CI 0.07, 0.47, *p* = 0.0005). This systematic review shows that tubeless PCNL can be safely performed and the standout benefits are shorter operative time and hospital stay, and a lower rate of postoperative urinary fistula.

**Supplementary Information:**

The online version contains supplementary material available at 10.1007/s00240-022-01337-y.

## Introduction

The eternal debate for percutaneous nephrolithotomy (PCNL) exit strategy is whether a nephrostomy tube is necessary and its impact on the procedure and complications. Furthermore, the presence of a nephrostomy tube may hamper PCNL as a day surgery/ambulatory procedure. PCNL access and exit strategies have been well defined by CROES and large-volume randomized controlled trials [1]. Exit strategies in PCNL are typically tubeless (refers to the placement of a double J stent alone), totally tubeless (refers to no nephrostomy and no double J stent), and nephrostomy alone [2]. There is a lack of consensus on what measurable intraoperative and postoperative outcomes, including exit strategy, are best suited for a day surgery/ambulatory PCNL. Tubeless PCNL could be the ideal approach in selecting which patients might be suitable for same-day discharge.

Three past systematic reviews favored tubeless PCNL over PCNL with a nephrostomy tube as it significantly shortened hospital stay allowing for a faster return to normal activity facilitated by lower immediate postoperative pain scores, reduced analgesic requirement, and urine leakage [2–4]. However, questions persist if this approach could increase complications rates, such as bleeding, urinomas, or perinephric abscess as well as hospital readmissions. The present study aims to evaluate how recent technical and technological refinements in PCNL have influenced urologists toward a clearer understanding of which choices, surgical outcomes, and parameters can be considered for PCNL, especially when planning their exit strategy with the usage of a nephrostomy tube placement vis-a-vis a tubeless approach.

## Methods

### Aim of the review

The present study aims to systematically review the safety and stone-free rate after tubeless PCNL (ureteral stent/ureteral catheter and no nephrostomy tube) as compared to standard PCNL (with nephrostomy tube with or without ureteral stent/ureteral catheter) for kidney stones. The main outcome is to evaluate for differences in surgical time, length of stay, and postoperative complications between two procedures. The secondary outcome is to assess if there is any difference in the stone-free rate (SFR) between the two procedures. We also intend to observe if there were any specific trends in tract sizes over the years as well as the use of different exit strategies in the tubeless cohort. Finally, we aim to compare the results of our meta-analysis to those of previous years.

### Literature search

This study was performed according to the 2020 Preferred Reporting Items for Systematic Reviews and Meta-Analyses (PRISMA) framework. A broad literature search was performed on 5th October 2021, using EMBASE, MEDLINE, and Cochrane Central Controlled Register of Trials (CENTRAL). Medical Subject Heading (MeSH) terms and keywords such as “kidney calculi”, “urolithiasis”, “Percutaneous Nephrolithotomy”, “PCNL”, “percutaneous lithotripsy”, “JJ or double J or pigtail or stent or catheter, “tubeless”, or "no tube" were used. No date limits were imposed. The search was restricted to English papers only. Animal and pediatric studies were excluded. Appendix shows the search strategy. Additional articles were sought from the reference lists of the included articles. This review was registered in PROSPERO (CRD42021291272).

### Selection criteria

The PICOS (Patient Intervention Comparison Outcome Study type) model was used to frame and answer the clinical question. P: adults undergoing PCNL for kidney stones; Intervention: standard PCNL (with nephrostomy tube with or without ureteral stents); Comparison: tubeless PCNL (no nephrostomy tube with ureteral stent); Outcome: surgical time, length of postoperative stay, infection complications (fever defined as body temperature > 38 °C, urinary tract infection, sepsis), bleeding complications (hemoglobin drop, blood transfusion, need for angioembolization rates), postoperative pain (visual analogue scale (VAS) score at fist postoperative day, patients requiring pain medication), urinary fistula (urinary leakage that may necessitate secondary drainage), perirenal fluid collection, pleural breach, hospital readmission for any reason and stone-free rate; Study type: prospective randomized studies. Patients were assigned to two groups according to the type of mode of exit strategy after PCNL (Tubeless PCNL vs Standard PCNL).

### Study screening and selection

Two independent authors screened all retrieved records through Covidence Systematic Review Management^®^ (Veritas Health Innovation, Melbourne, Australia). A third author solved discrepancies. Studies were included based on PICOS eligibility criteria. Prospective randomized studies were accepted. Retrospective and prospective nonrandomized studies, reviews, meeting abstracts, letters to the editor, case reports, and editorials were excluded. The full text of the screened papers was selected if found relevant to the purpose of this study. The search was further expanded by performing a manual search based on the references of the full-text relevant papers.

### Statistical analysis

Surgical time, hemoglobin drop, postoperative length of stay, and VAS score were pooled using the inverse variance of the mean difference with a random effect, 95% confidence interval (CI), and *p* values. Incidence of blood transfusion, angioembolization for bleeding control, patients requiring postoperative pain medication, postoperative infection complications, urinary fistula, perirenal fluid collection, pleural breach, readmission, and stone-free rate were assessed using Cochran–Mantel–Haenszel Method with the random effect model and reported as risk ratio (RR), 95% CI, and *p* values. Analyses were two-tailed and the significance was set at *p* < 0.05 and a 95% CI. OR less than one indicates a lower risk in the tubeless group. Study heterogeneity was assessed utilizing the *I*^2^ value. Substantial heterogeneity was defined as an *I*^2^ value > 50%. Meta-analysis was performed using Review Manager (RevMan) 5.4 software by Cochrane Collaboration. The quality assessment of the included studies was performed using the RoB 2 Cochrane Risk of Bias tool.

## Results

Literature search retrieved 1424 papers. Three papers were found from other sources. Thirty-two duplicates were excluded, leaving 1395 studies for screening. Another 1266 papers unrelated to the study purpose were further excluded after the title and abstract screening. The full texts of the remaining 129 studies were screened and 103 papers were further excluded. Finally, 26 studies were accepted and included for meta-analysis. Supplementary Fig. 1 shows the 2020 PRISMA flow diagram.

### Study characteristics and quality assessment

Twenty-six randomized studies compared Tubeless and Standard PCNL [5–30]. Study characteristics are summarized in Table 1. There were 1839 patients included in 26 studies: 907 patients underwent Tubeless PCNL and 932 underwent Standard PCNL.

**Table 1:** Characteristics of studies comparing Tubeless PCNL vs Standard PCNL included in the review

Author year of publication	Tract dilatation	Amplatz sheath size, Fr	Nephrostomy tube size, Fr	Stent size, Fr	Tract closure	Stent dwelling time	Nephrostomy dwelling time	Definition of stone-free	Mean age Tubeless PCNL, years	Mean age Standard PCNL, years	Mean stone burden Tubeless PCNL	Mean stone burden Standard PCNL
Agrawal 2008 [5]	NA	26	16	6	Suture	2 weeks	2–3 days	NA	33	31	3.8 cm^2^	3.6 cm^2^
Ali 2019 [6]	Alken coaxial metallic dilators	26	NA	NA	no	NA	NA	NA	44.04	44.24	NA	NA
Bhat 2017 [7]	One-shot 30 Ch Amplatz dilator	30	22	NA	no	NA	NA	NA	NA	NA	NA	NA
Chalise, 2017 [8]	NA	NA	20–24	NA	Suture	NA	36–48 h	NA	37.1	37.9	23 mm	23.7 mm
Choi 2006 [9]	Serial metal dilators	34	8.2	6	Gel matrix thrombin	3–5 Days if no significant residual fragments	3–5 Days if no significant residual fragments	No fragments	52.9	47	28.5 mm	26.8 mm
Cormio 2012 [10]	Balloon/plastic dilators	30	16	7	Tachoseal	1 day	3 days	4 mm or less	51.38	49.24	32.17 mm	30.22 mm
Desai 2004 [11]	Alken metal dilators	26–30	20	6	No	4 weeks	2 days	NA	43.4	41.1	25 mm^3^	264 mm^3^
Etemadian 2011 [12]	One-shot Amplatz dilator	30	24	NA	No	24–48 h	24–48 h	NA	44.58	46.55	36.26 mm	35.35 mm
Feng 2001 [13]	Amplatz dilators	34	22	NA	NA	1 week	2 days	No stones	62	53	4.38 cm2	8.36 cm^2^
Garg 2019 [14]	NA	NA	NA	NA	NA			NA	36.78	36.78	NA	NA
Goldberg 2020 [15]	Balloon	30	12	6	NA	2 weeks	2 days	≤ 3 mm	55.37	55.38	23 mm	24.2 mm
Gonen 2019 [16]	Amplatz dilator	30	14	6	NA	24 h	2 days	NA	47.5	45	35.73 mm^3^	386.2 mm^3^
Jiang 2017 [17]	NA	NA	18	5	NA	2 weeks	2 days	NA	45.9	48.1	166 mm^3^	189.7 mm^3^
Kara 2020 [18]	NA	28	18	NA	NA	24 h	3–5 days	NA	67.7	66.5	25.6 mm	22.3 mm
Kirac 2013 [19]	NA	30	14	NA	NA	14–21 days	1–3 days	No fragments	43.5	42.5	25.4 mm	30.5 mm
Kumar 2020 [20]	NA	26	20	NA	no	NA	NA	NA	NA	NA	NA	NA
Liu 2017 [21]	NA	16–18	16	6	NA	4 weeks	4–5 days	< 4 mm	46.08	48.6	1.98 cm	1.82 cm
Marchant 2011 [22]	Fascial dilators	28	18	7	Oxidized cellulose gauze	10–14 days	3 days	NA	52.8		6.4 cm^2^	7.8 cm^2^
Mishra 2010 [23]	Metallic telescopic coaxial dilators	28	20	NA	manual compression for 5 min	24 h If no re-treatment was planned	24 h If no re-treatment was planned	No evidence of clinically insignificant residual fragments on CT	55	50	NA	NA
Sebaey 2016 [24]	14-F Teflon	14	14	NA	Suture	2–3 days	NA	NA	48.4	50.1	182 mm^3^	191mm^3^
Shah 2008 [25]	Telescopic dilators	30	8	6	Strapped with a pressure dressing	Until clearing urine	24 h	< 4 mm	48.66	42.08	535.3 mm^2^	495.91 mm^2^
Shoma 2011 [26]	NA	NA	22	NA	Suture	Until clearing urine	Until clearing urine	Residual stones of 4 mm	31	34	1226 mm^2^	1004 mm^2^
Singh 2008 [27]	NA	NA	22	NA	NA	NA	NA	NA	52.6	55.2	750 mm^2^	800 mm^2^
Sofikerim 2007 [28]	NA	30	18	6	Suture	14 days	2 days	No stones	38.4	41.3	425 mm^2^	428 mm^2^
Tefekli 2007 [29]	NA	30	14	NA	Suture	24 h	48 h	No stones	58	57.4	300 mm^2^	310 mm^2^
Zhao 2016 [30]	NA	30	12	NA	Suture or fibrine sealant	2 weeks	48 h	No stones	48.88	53.05	259 mm^2^	276.6 mm^2^

*Fr* French, *PCNL* percutaneous nephrolithotomy, *NA* not available

Supplementary Fig. 2 demonstrates the details of the quality assessment of included studies. Fourteen studies showed a low overall risk of bias. Ten studies showed some concerns regarding the overall risk of bias and the remaining two studies a high overall risk of bias. The most frequent reason for bias was bias due to deviation of the intended intervention and measurement outcomes, followed by bias due to the randomization process.

### Meta-analyses of surgical time and length of stay

**Fig. 1:**
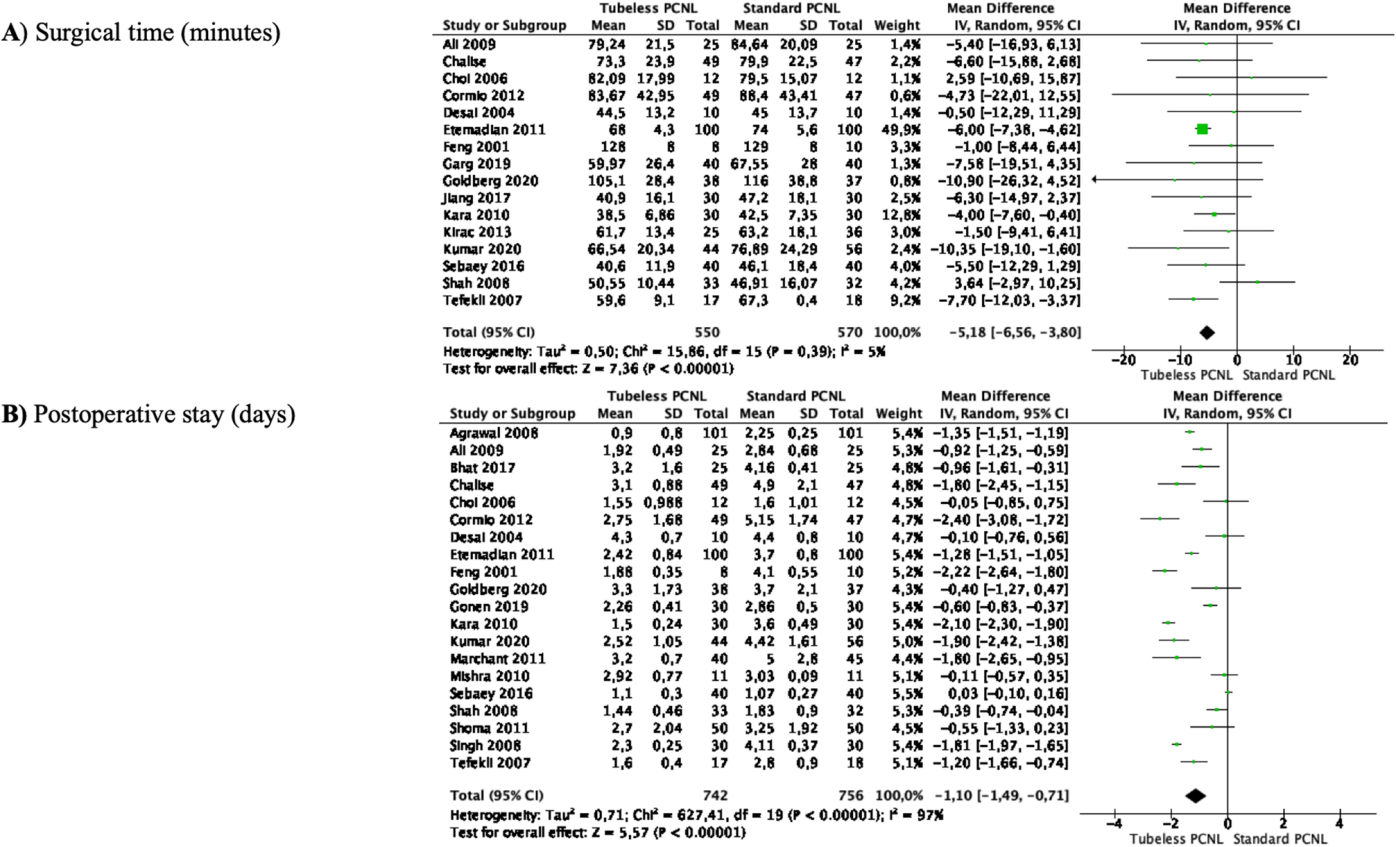
Meta-analysis of surgical time and length of stay in studies comparing Tubeless PCNL vs Standard PCNL

Meta-analysis from 16 studies (550 cases in Tubeless and 570 cases in Standard PCNL) showed that the mean operative time was significantly shorter in Tubeless compared to Standard PCNL (MD—5.18 min, 95% CI − 6.56 to − 3.80, *p* < 0.00001). There was no significant heterogeneity among the studies (*I*^2^ 5%) (Fig. 1A).

Meta-analysis of 20 studies (742 cases in Tubeless and 756 cases in Standard PCNL) showed that the mean postoperative length of stay was also significantly shorter in Tubeless compared to Standard PCNL (MD—1.10 days, 95% CI − 1.48 to − 0.71, *p* < 0.00001). Study heterogeneity was considerable (*I*^2^ 97%) (Fig. 1B).

### Meta-analyses of bleeding

**Fig. 2:**
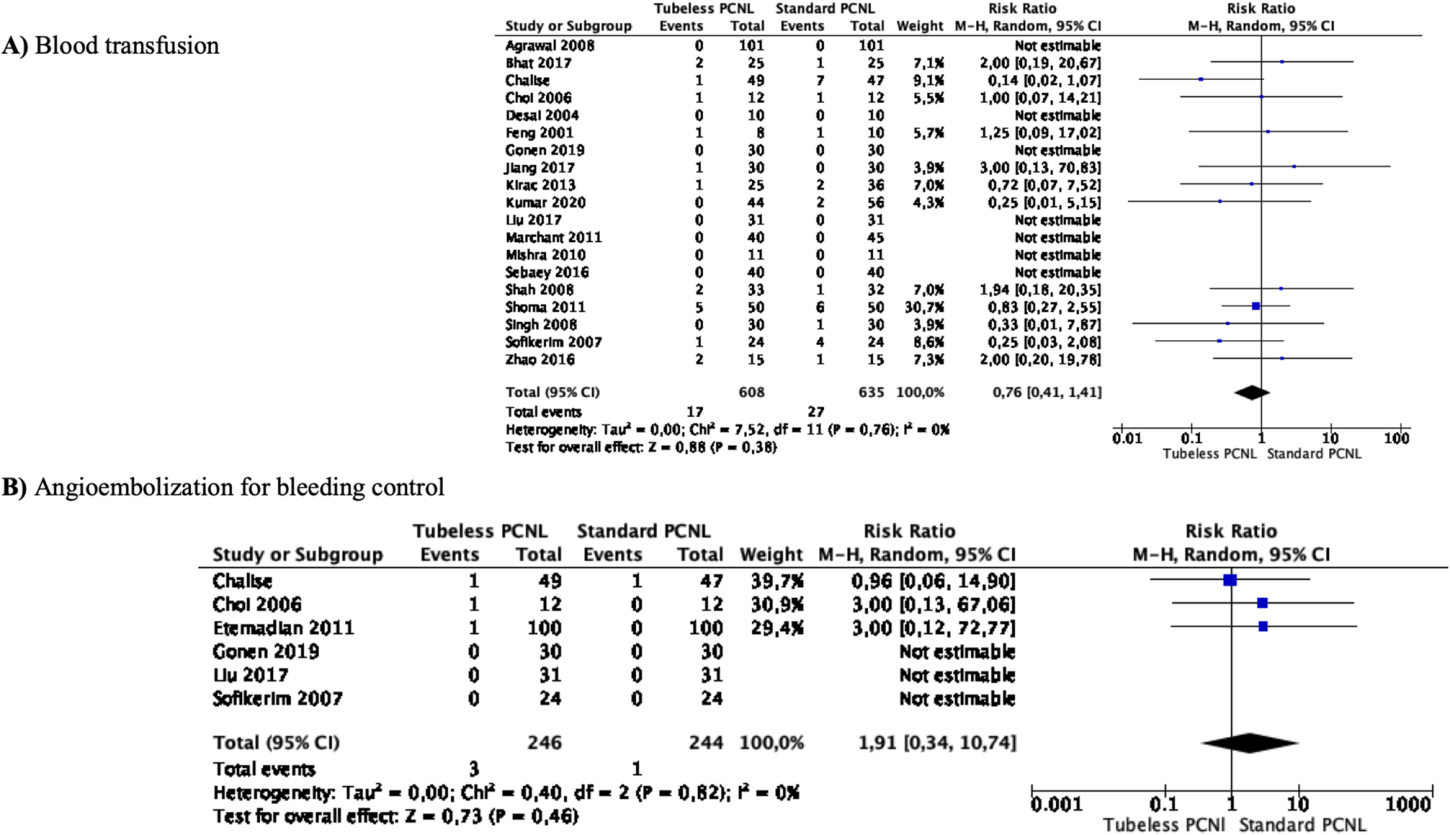
Meta-analysis of bleeding in studies comparing Tubeless PCNL vs Standard PCNL

Meta-analysis from 19 studies (608 cases in Tubeless PCNL and 635 cases in Standard PCNL) showed that blood transfusion did not differ between the two groups (RR 0.76 95% CI 0. 14–1.41, *p* = 0.38). There was no significant heterogeneity among the studies (*I*^2^ 0%) (Fig. 2A).

Meta-analysis from 6 studies (246 cases in Tubeless PCNL and 244 cases in Standard PCNL) showed that angioembolization for bleeding control did not differ between the two groups (RR 1.91 95% CI 0.34–10.74, *p* = 0.46). There was no significant heterogeneity among the studies (*I*^2^ 0%) (Fig. 2B).

### Meta-analyses of postoperative pain

**Fig. 3:**
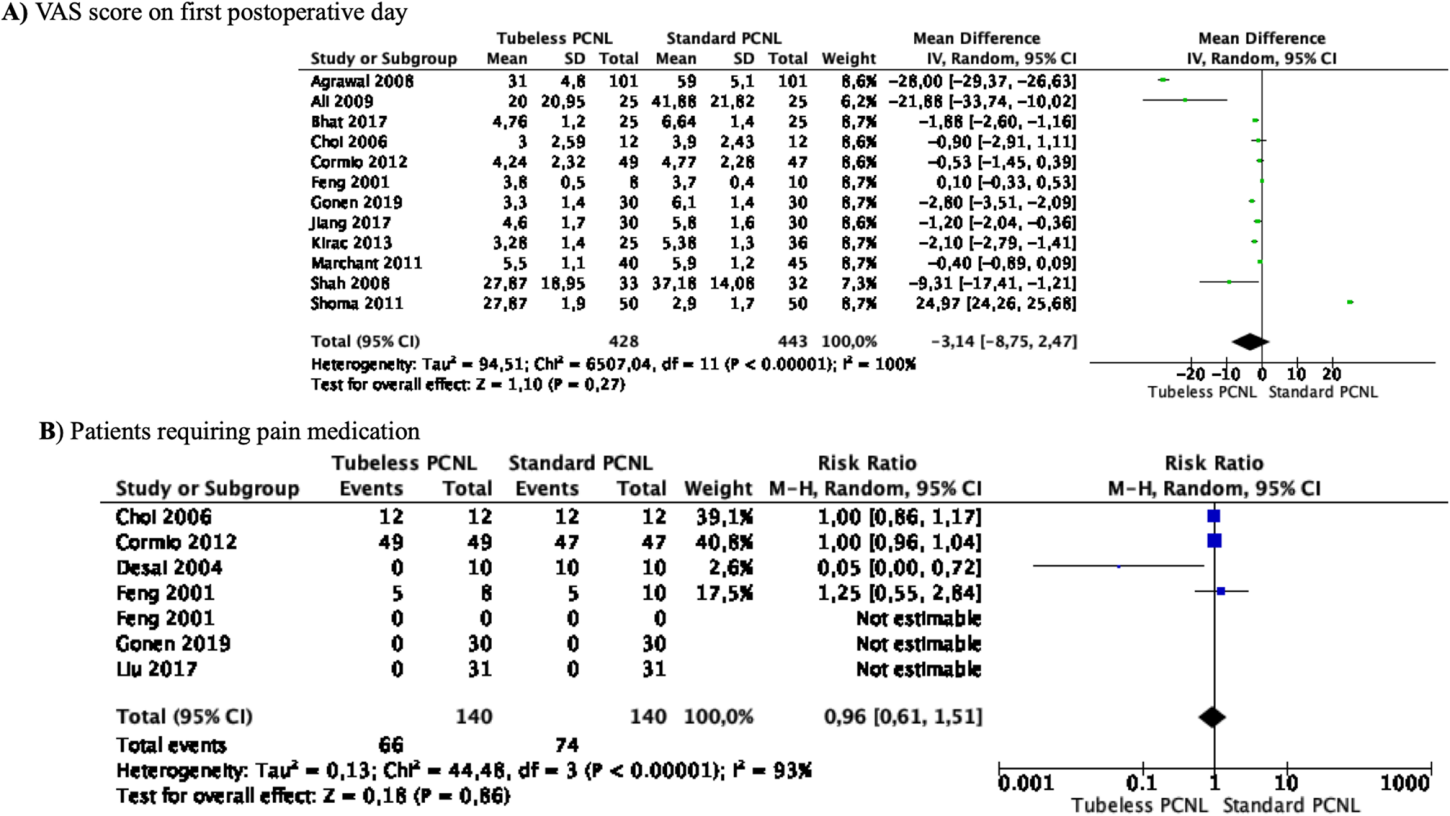
Meta-analysis of postoperative pain in studies comparing Tubeless PCNL vs Standard PCNL

Meta-analysis from 12 studies (428 cases in Tubeless PCNL and 443 cases in Standard PCNL) showed that the mean VAS score at the first postoperative day did not differ between the two groups (MD—3.14 points 95% CI − 8.75 to 2.47, *p* = 0.27) (Fig. 3A). Study heterogeneity was considerable (*I*^2^ 100%).

Meta-analysis from 7 studies (140 cases in Tubeless PCNL and 140 cases in Standard PCNL) showed that patients requiring postoperative pain medication did not differ between the two groups (RR 0.96 95% CI 0.61–1.51, *p* = 0.86) (Fig. 3B). Heterogeneity among the studies was significant (*I*^2^ 93%).

### Meta-analyses of infection complications

**Fig. 4:**
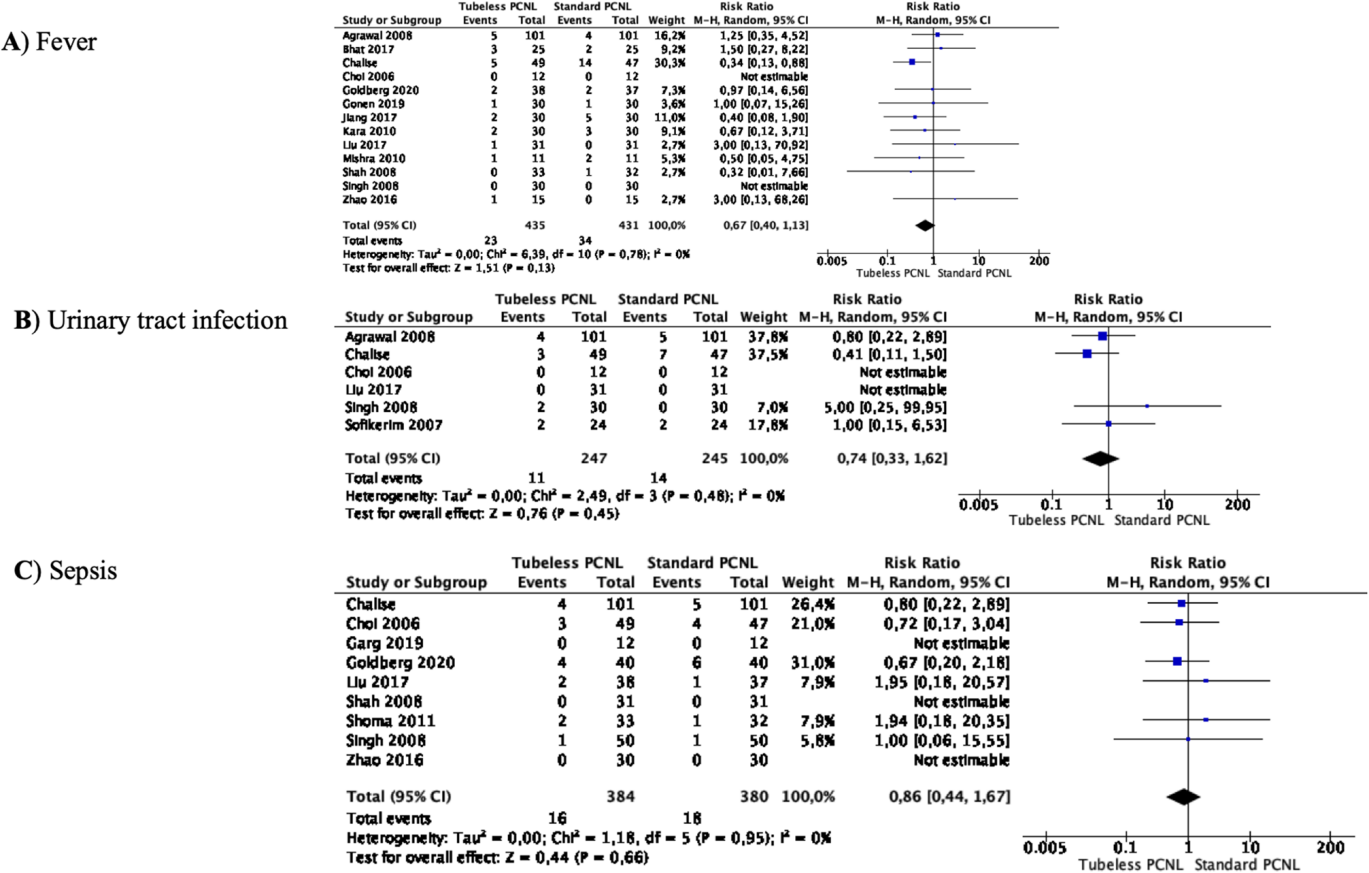
Meta-analysis of infection complications in studies comparing Tubeless PCNL vs Standard PCNL

Meta-analysis from 13 studies (435 cases in Tubeless PCNL and 431 cases in Standard PCNL) showed that the incidence of postoperative fever did not differ between the two groups (RR 0.67 95% CI 0.40–1.13, *p* = 0.13) (Fig. 4A). There was no significant heterogeneity among the studies (*I*^2^ 0%).

Meta-analysis from 6 studies (247 cases in Tubeless PCNL and 245 cases in Standard PCNL) showed that the incidence of postoperative urinary tract infection did not differ between the two groups (RR 0.74 95% CI 0.33–1.62, *p* = 0.45) (Fig. 4B). There was no significant heterogeneity among the studies (*I*^2^ 0%).

Meta-analysis from 9 studies (384 cases in Tubeless PCNL and 380 cases in Standard PCNL) showed that the incidence of postoperative sepsis did not differ between the two groups (RR 0.86 95% CI 0.44–1.67, *p* = 0.66) (Fig. 4C). There was no significant heterogeneity among the studies (*I*^2^ 0%).

### Meta-analyses of urinary fistula, perirenal fluid collection, and pleural breach

**Fig. 5:**
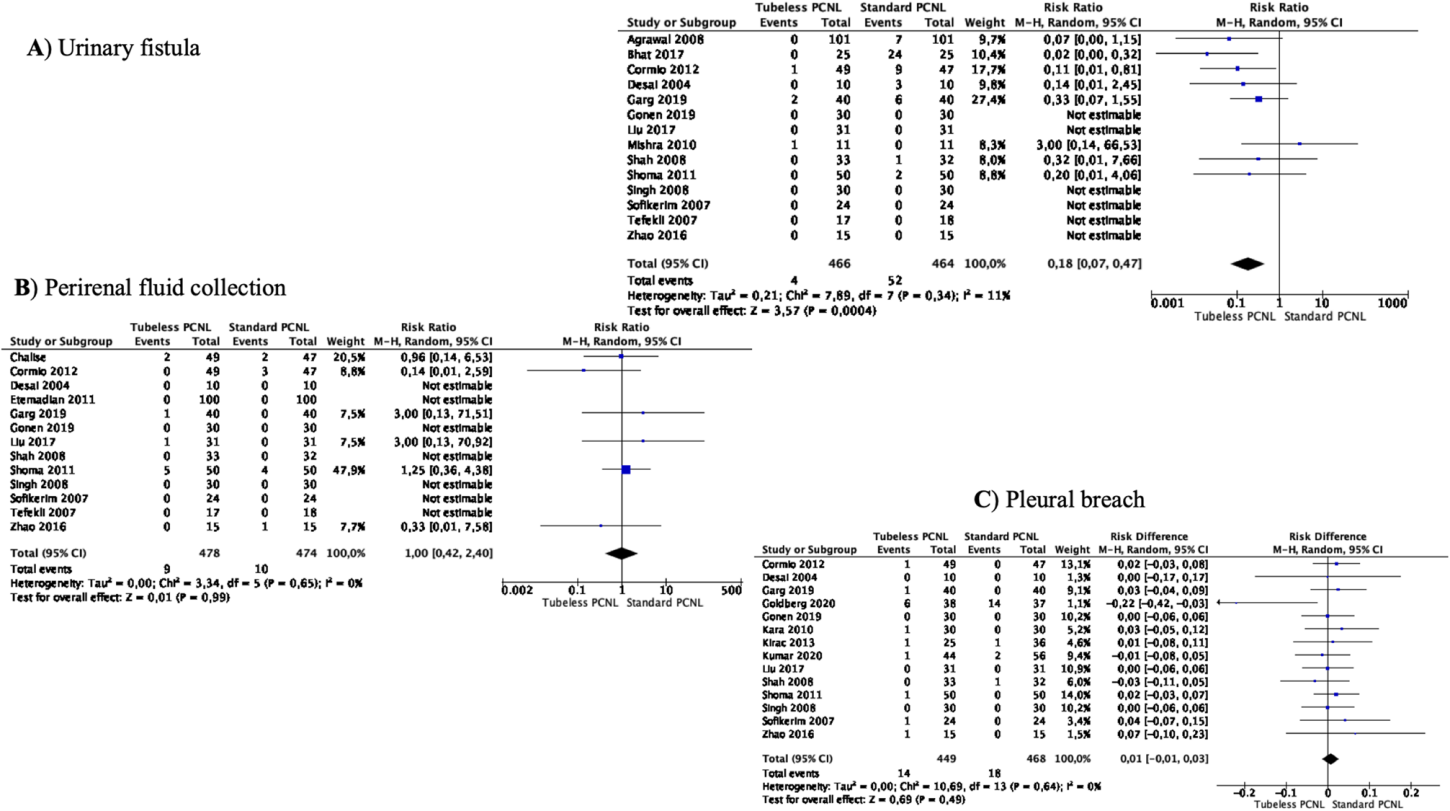
Meta-analysis of urinary fistula, perirenal fluid collection, and pleural breach in studies comparing Tubeless PCNL vs Standard PCNL

Meta-analysis from 14 studies (466 cases in Tubeless PCNL and 464 cases in Standard PCNL) showed that the incidence of the postoperative urinary fistula was significantly lower in the Tubeless PCNL group (RR 0.18 95% CI 0.07–0.47, *p* = 0.0005) (Fig. 5A). Heterogeneity among the studies was not important (*I*^2^ 11%).

Meta-analysis from 13 studies (478 cases in Tubeless PCNL and 474 cases in Standard PCNL) showed that the incidence of the postoperative perirenal fluid collection did not differ between the two groups (RR 1.00 95% CI 0.42–2.40, *p* = 0.99) (Fig. 5B). There was no significant heterogeneity among the studies (*I*^2^ 0%).

Meta-analysis from 14 studies (449 cases in Tubeless PCNL and 468 cases in Standard PCNL) showed that the incidence of pleural breach did not differ between the two groups (RR 0.01 95% CI -0.01–0.03, *p* = 0.49) (Fig. 5C). There was no significant heterogeneity among the studies (*I*^2^ 0%).

### Meta-analyses of hospital readmission and stone-free rate

**Fig. 6:**
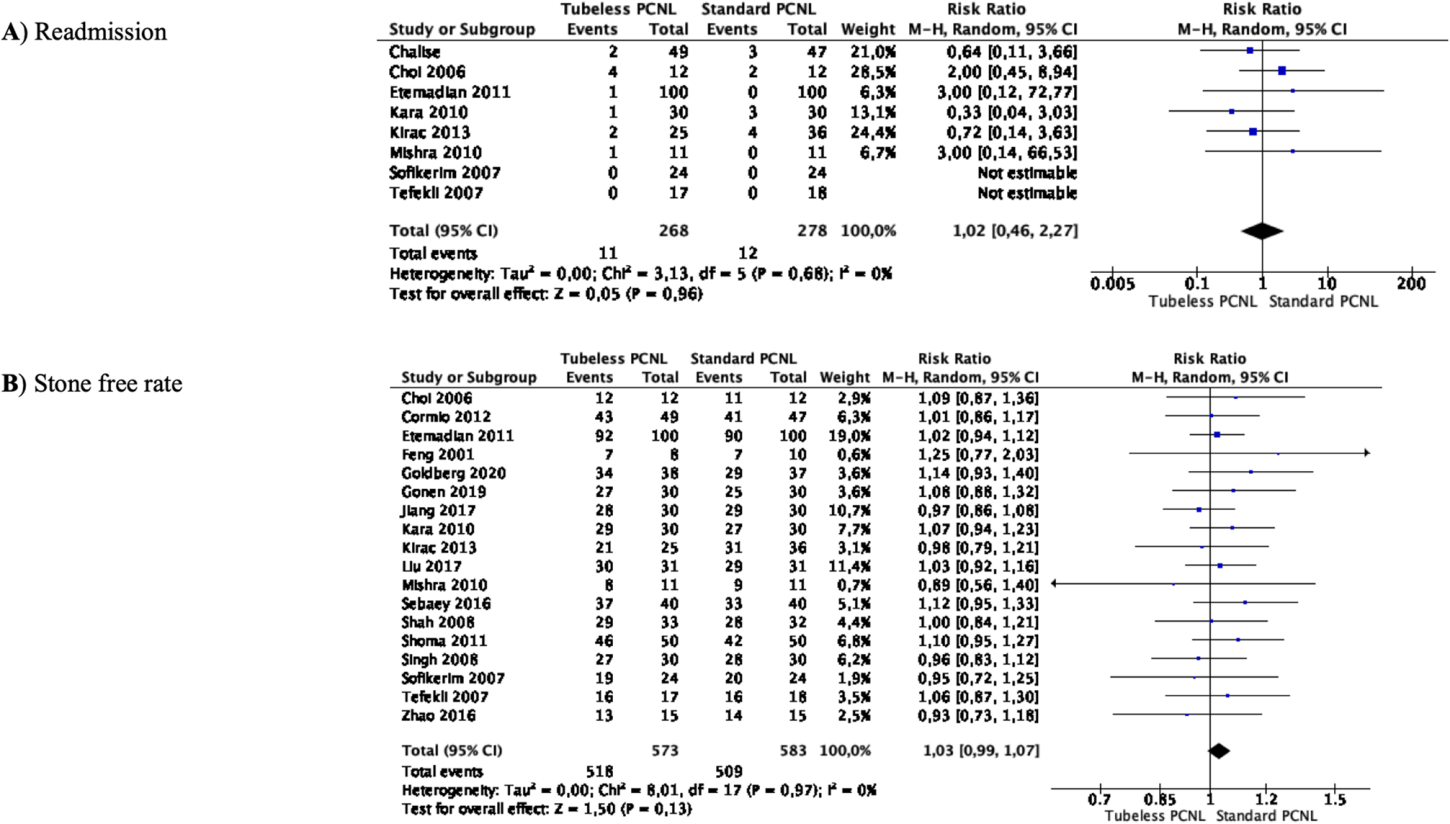
Meta-analysis of hospital readmission for any reasons and stone-free rate in studies comparing Tubeless PCNL vs Standard PCNL

Meta-analysis from 8 studies (268 cases in Tubeless PCNL and 278 cases in Standard PCNL) showed that the incidence of hospital readmission for any reason did not significantly differ between the two groups (RR 1.02 95% CI 0.46–2.27, *p* = 0.96) (Fig. 6A). There was no significant heterogeneity among the studies (*I*^2^ 0%).

Meta-analysis from 18 studies (573 cases in Tubeless PCNL and 583 cases in Standard PCNL) showed that the stone-free rate did not differ between the groups (RR 1.03 95% CI 0.99–1.07, *p* = 0.13) (Fig. 6B). There was no significant heterogeneity among the studies (*I*^2^ 0%).

## Discussion

PCNL was first described in 1976 by Fernström and Johansson, and has evolved and revolutionized the way renal calculi are managed [31] and is the current standard in stones larger than 2 cm across international guidelines [32, 33]. In 1999, Goh et al. described PCNL using a 30–34 Ch Amplatz sheath with this trend carrying into the early 2000s [34]. In 2007, Brusky et al. performed mini-PCNL through a 20 Ch percutaneous access starting the trend for miniaturization in PCNL [35]. After initial skepticism, miniaturized PCNL is now an accepted way forward with tract sizes dropping from 30 to 24 Fr for a standard PCNL and sizes less than 20 Fr being considered a mini-PCNL surgery [36].

This led to the belief that miniaturized PCNL should ideally replace standard PCNL; however, as seen in our review, many centers continue using standard access in both arms of their study (Supplementary Fig. 3). Our study is the only systematic review that has looked at tract size, and only two studies out of 26 studies used a 16 Fr and 14 Fr tract, respectively [21, 24]. Postprocedure, the most common nephrostomy tube placed was 20 Fr and above. Only two studies placed an 8 Fr nephrostomy tube as an exit in a 34 Fr tract [9, 25]. Supplementary Fig. 3 shows the correlation between the tract size and the nephrostomy tube used in studies included in this review.

In our analysis, no specific trends were noted between tract size and nephrostomy tube size based on stone volume. Most series were comparable in the stone volume included in both groups, and these often included partial and complete staghorn stones. However, the specific number of these cases was not available in individual studies to consider a subset analysis to see if this has any impact on outcomes.

Our findings suggest that a tailored and personalized approach is the need of the day for modern PCNL, and urologists should not shy away from using bigger tracts to achieve a good outcome [37]. Significant advancements in miniaturization [36], adoption of new positions [38], and technological enhancements have provided a plethora of choices for lithotripsy, ranging from ballistic to laser to combined energy devices [39]. The aforementioned improvements tackle any stone composition and volume, with minimal complications and maximum efficacy, making PCNL feasible for a day surgery/ambulatory procedure in a select group of patients.

The first work about tubeless PCNL was published in 1997 by Bellman et al. without significant complications and early discharge of all 50 patients [40]. Limb et al. tried to specify discharge criteria to objectively compare the length of stay between standard and tubeless PCNL [41]. They reported different factors that may bias this variable such as health care system policies, patient’s concomitant morbidities, and the variability of subjective pain assessment. Albeit limited, evidence from the past on this topic has shown that these are the key areas to consider for any PCNL outcome apart from stone and patient characteristics [2–4, 42]. Our review confirmed that the mean hospital stay (MD—1.10 days, 95% CI − 1.48 to − 0.71, *p* < 0.00001) and shorter operative time significantly favored the tubeless PCNL group (MD—5.18 min, 95% CI − 6.56 to − 3.80, *p* < 0.00001). However, the SFR in our analysis, defined in most studies as residual fragments < 4 mm and/or no fragment seen on table inspection or at first imaging, did not differ between the two groups. Generally, a 100% SFR is preferable for PCNL, but this is not a reason to delay or postpone hospital discharge. Since the SFR did not differ, it could be interpreted that achieving a 100% SFR should not be a precluding factor for a tubeless PCNL.

With regards to postoperative pain and analgesic requirements, similar or better outcomes favored tubeless PCNL in previous systematic reviews [2–4, 43]. Maheshwari et al. proposed that even in a Standard PCNL by just leaving a small pigtail in situ as a nephrostomy tube, patients can be discharged earlier, pain score is better, complications are less, and recovery is faster [44]. Interestingly, Eslahi et al. also found that the amount of narcotic use and pain were significantly lower in totally tubeless PCNL (no ureteral stent and no nephrostomy tube) as compared with standard and tubeless PCNL [45]. Our meta-analysis on postoperative pain favored the tubeless cohort; however, it was not statistically significant. Furthermore, the need for postoperative pain medication was not different between the two groups. This reiterates the concept that tubes probably have minor effects on postoperative pain. We could not identify if intraoperative tract infiltration for pain management is now a common trend, but this could aid in immediate postoperative pain management and analgesic requirements, especially if a patient is being considered for same-day discharge [25]. Same-day discharge PCNL is indeed a reality today as shown recently by a 500-patient study by Chong et al., where they reported the use of a standard tract dilation (24–30 Fr) in 77% of cases, and 99% of cases had a ureteral stent as the only form of drainage [46]. However, 2.4% required early readmission, and the 30-day readmission rate was 4.2% [46]. While systematic reviews in the past have shown that Tubeless PCNL allowed for a faster return to work, and this is an important aspect to take into account if patients are getting readmitted which defeats the purpose of a day surgery [2, 4].

Complications are a dreaded part of PCNL. In the past systematic reviews, no statistical differences for hemoglobin drop and blood transfusion were seen [3, 4]. In our analysis, 19 studies did not show any difference in the rate of blood transfusion, and in the 6 studies that reported incidence of angioembolization post-PCNL, no absolute difference was noted. Most studies in our review had tract size greater than 20 Fr and serial dilatation was the preferred approach in 10 of the 13 reported series. These findings have practical utility for urologists while planning a desired tract size and technique of renal access. Complications like fever, sepsis, urinary infections, pleural injury, and incidence of perirenal fluid collections did not differ between the two groups in our analysis, but the incidence of postoperative urinary fistula was significantly lower in the Tubeless PCNL group (RR 0.18 95% CI 0.07–0.47, *p* = 0.0005). There was no trend noted for using sealants and only 4 of the 26 studies used some tract sealants, but the use of sealants disappeared after 2016. Another important finding of our meta-analysis was that the incidence of hospital readmission for any reason did not significantly differ between the two groups (RR 1.02 95% CI 0.46–2.27, *p* = 0.96). This is very important as it allows urologists full flexibility in choosing any approach feasible to their realm of practice and is the quintessential for counseling patients during preoperative planning.

While our review has the inherent bias associated with patient selection that may vary across the world, we have a significantly higher number of included studies as compared to any past similar meta-analysis, and this allows us to dive deeper and compare the pros and cons of Tubeless PCNL vis-a-vis Standard PCNL. Hence, this could account for the differences from other reviews. The lack of reported outcomes such as the number of staghorn stones prevented any form of subset analysis. We also could not perform a cost analysis and assess quality of life of patients.

## Conclusions

Our review shows that the standout benefits of Tubeless PCNL are shorter operative time, shorter hospital stay, and a lower rate of postoperative urinary fistula. However, pain scores, need for readmission, use of analgesia, and complication rates did not differ between the groups, making Tubeless PCNL a safe option that deserves further studies to assess its role in a same-day discharge approach.

## Supplementary Information

Below is the link to the electronic supplementary material.

Supplementary file1 (DOCX 18 KB)

Supplementary file2 (DOCX 48 KB)

Supplementary file3 (DOCX 468 KB)

Supplementary file4 (DOCX 31 KB)
