# Supplementary material for: Complications and outcomes of tubeless versus nephrostomy tube in percutaneous nephrolithotomy: a systematic review and meta-analysis of randomized clinical trials
Source: Urolithiasis. 2022 Jun 8;50(5):511–22. doi: 10.1007/s00240-022-01337-y (PMC9468100; doi:10.1007/s00240-022-01337-y)
Supplement: Supplementary file 1 — Supplementary file1 (DOCX 18 KB) [file 240_2022_1337_MOESM1_ESM.docx]

**Appendix:** literature search strategy.

Database: Embase <1980 to 2021 Week 39>, EBM Reviews - Cochrane Central Register of Controlled Trials <August 2021>, Ovid MEDLINE(R) ALL <1946 to October 05, 2021>

Search Strategy:

--------------------------------------------------------------------------------

1 exp kidney calculi/ or exp urolithiasis/ (103444)

2 (((Kidney or renal or staghorn or nephro*) adj3 (stone* or calcul* or lithiasis)) or nephroliasis or urolithiasis).tw,kw. (65104)

3 exp Nephrolithotomy, Percutaneous/ (9522)

4 (Percutaneous Nephrolithotomy or PCNL or percutaneous lithotripsy).tw,kw. (14885)

5 3 or 4 (16338)

6 (((kidney or renal or urethr* or JJ or double J or pigtail) adj3 (stent* or catheter)) or tubeless or "no tube").tw,kw. (42074)

7 5 and 6 (2175)

8 (child/ or Pediatrics/ or Adolescent/ or Infant/ or adolescence/ or newborn/ or (baby or babies or child or children or pediatric* or paediatric* or peadiatric* or infant* or infancy or neonat* or newborn* or new born* or adolescen* or preschool or pre-school or toddler*).tw.) not (adult/ or aged/ or (aged or adult* or elder* or senior* or men or women).tw.) (4453597)

9 (exp animals/ or exp animal/ or exp nonhuman/ or exp animal experiment/ or animal model/ or animal tissue/ or non human/ or (rat or rats or mice or mouse or swine or porcine or murine or sheep or lambs or pigs or piglets or rabbit or rabbits or cat or cats or dog or dogs or cattle or bovine or monkey or monkeys or trout or marmoset$1).ti.) not (humans/ or human/ or human experiment/ or (human* or men or women or patients or subjects).tw.) (10324964)

10 8 or 9 (14567293)

11 7 not 10 (2031)

12 remove duplicates from 11 (1424)

***************************
