# Supplementary material for: Complications and outcomes of tubeless versus nephrostomy tube in percutaneous nephrolithotomy: a systematic review and meta-analysis of randomized clinical trials
Source: Urolithiasis. 2022 Jun 8;50(5):511–22. doi: 10.1007/s00240-022-01337-y (PMC9468100; doi:10.1007/s00240-022-01337-y)
Supplement: Supplementary file 2 — Supplementary file2 (DOCX 48 KB) [file 240_2022_1337_MOESM2_ESM.docx]

**Identification of studies via databases and registers**

Records removed *before screening*:

Duplicate records removed (n = 32)

Records identified from:

Databases (n = 1415)

Registers (n = 9)

Other sources (n = 3)

**Identification**

Records screened

(n = 1395)

Records excluded (n = 1266)

Reports sought for retrieval

(n = 129)

Reports not retrieved

(n = 0)

**Screening**

Reports excluded (n = 103):

Meeting abstract (n = 59)

Review (n = 18)

Not in English (n = 5)

Prospective non randomized study (n = 5)

Duplicate study (n = 3)

Wrong patient population (n = 3)

Wrong study design (n = 3)

Editorial comment (n = 3)

Wrong outcomes (n = 2)

Wrong intervention (n = 1)

Wrong setting (n = 1)

Reports assessed for eligibility

(n = 129)

Studies included in review

(n = 26)

**Included**
