# Supplementary material for: Complications and outcomes of tubeless versus nephrostomy tube in percutaneous nephrolithotomy: a systematic review and meta-analysis of randomized clinical trials
Source: Urolithiasis. 2022 Jun 8;50(5):511–22. doi: 10.1007/s00240-022-01337-y (PMC9468100; doi:10.1007/s00240-022-01337-y)
Supplement: Supplementary file 3 — Supplementary file3 (DOCX 468 KB) [file 240_2022_1337_MOESM3_ESM.docx]

**Supplementary Figure 2.** Risk of bias in included studies (ROB-2)

1. Risk of bias graph: review authors' judgements about each risk of bias item presented as percentages across all included studies.


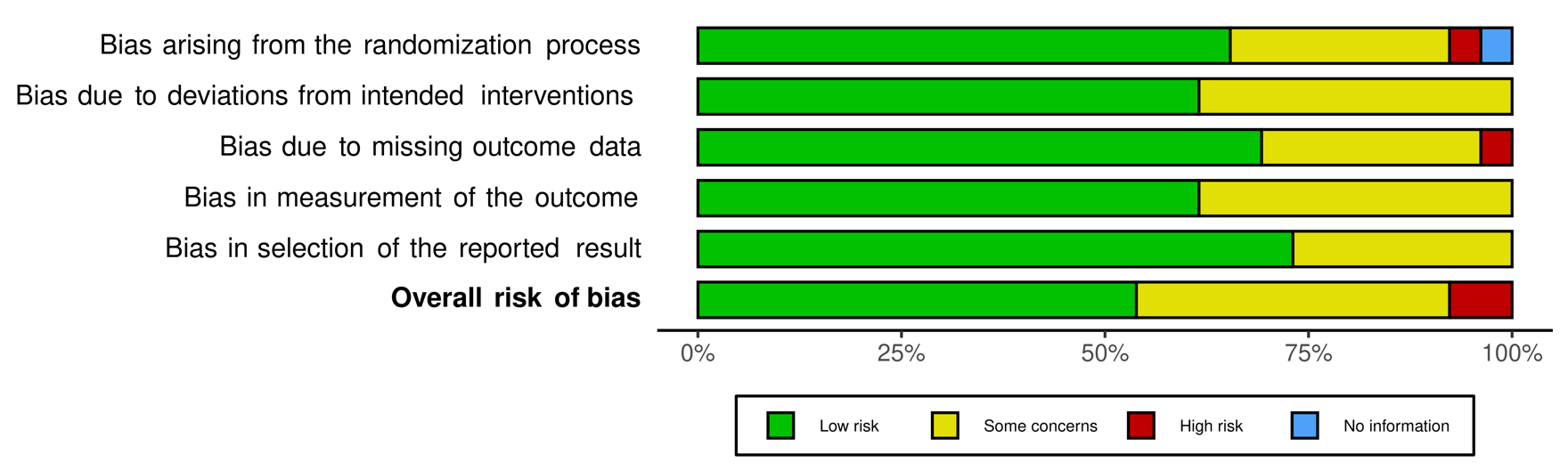


1. Risk of bias summary: review authors' judgements about each risk of bias item for each included study.

**
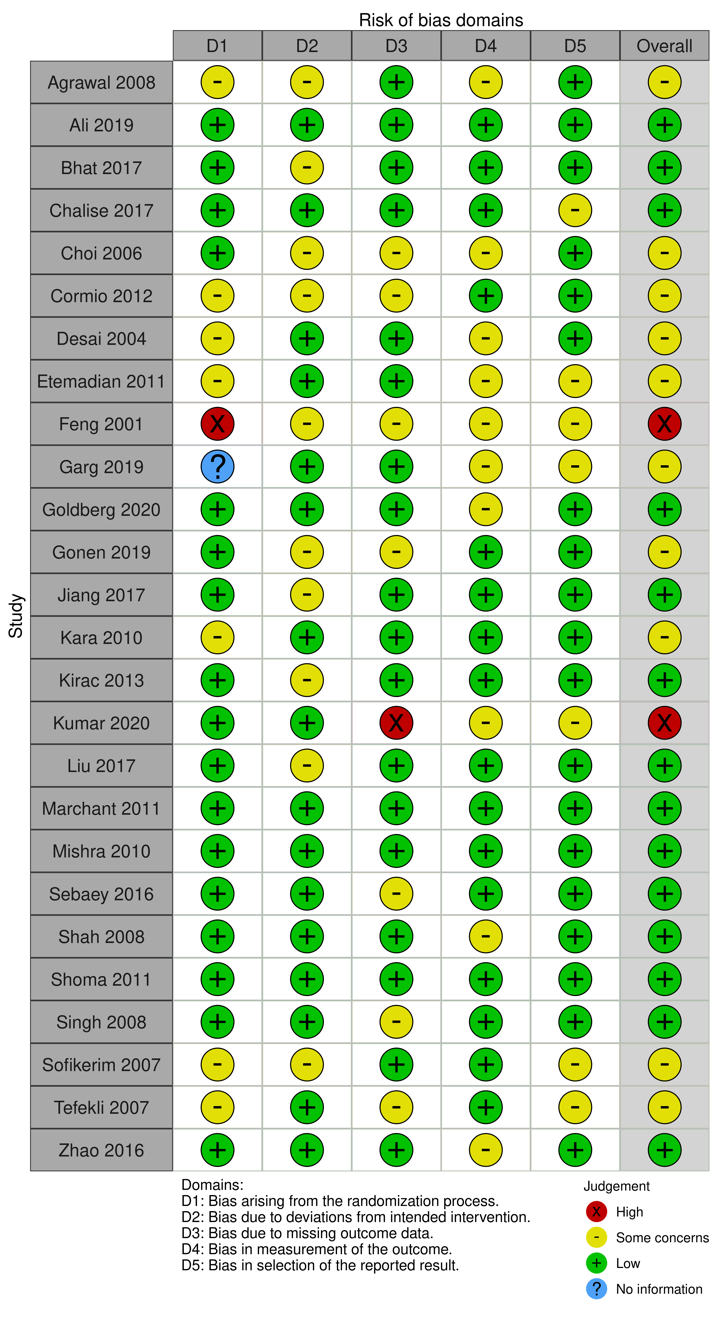
**
