# Supplementary material for: Complications and outcomes of tubeless versus nephrostomy tube in percutaneous nephrolithotomy: a systematic review and meta-analysis of randomized clinical trials
Source: Urolithiasis. 2022 Jun 8;50(5):511–22. doi: 10.1007/s00240-022-01337-y (PMC9468100; doi:10.1007/s00240-022-01337-y)
Supplement: Supplementary file 4 — Supplementary file4 (DOCX 31 KB) [file 240_2022_1337_MOESM4_ESM.docx]

**Supplementary Figure 3:** Trend in tract size (TS) and nephrostomy tube (NT) size over the years from studies included
